# *Brugia malayi* Excreted/Secreted Proteins at the Host/Parasite Interface: Stage- and Gender-Specific Proteomic Profiling

**DOI:** 10.1371/journal.pntd.0000410

**Published:** 2009-04-07

**Authors:** Sasisekhar Bennuru, Roshanak Semnani, Zhaojing Meng, Jose M. C. Ribeiro, Timothy D. Veenstra, Thomas B. Nutman

**Affiliations:** 1 Laboratory of Parasitic Diseases, National Institute of Allergy and Infectious Diseases, National Institutes of Health, Bethesda, Maryland, United States of America; 2 Laboratory of Proteomics and Analytical Technologies, SAIC-Frederick, National Cancer Institute at Frederick, Frederick, Maryland, United States of America; 3 Laboratory of Malaria and Vector Research, National Institute of Allergy and Infectious Diseases, National Institutes of Health, Bethesda, Maryland, United States of America; University of Pittsburgh, United States of America

## Abstract

Relatively little is known about the filarial proteins that interact with the human host. Although the filarial genome has recently been completed, protein profiles have been limited to only a few recombinants or purified proteins of interest. Here, we describe a large-scale proteomic analysis using microcapillary reverse-phase liquid chromatography-tandem-mass spectrometry to identify the excretory-secretory (ES) products of the L3, L3 to L4 molting ES, adult male, adult female, and microfilarial stages of the filarial parasite *Brugia malayi*. The analysis of the ES products from adult male, adult female, microfilariae (Mf), L3, and molting L3 larvae identified 852 proteins. Annotation suggests that the functional and component distribution was very similar across each of the stages studied; however, the Mf contributed a higher proportion to the total number of identified proteins than the other stages. Of the 852 proteins identified in the ES, only 229 had previous confirmatory expressed sequence tags (ESTs) in the available databases. Moreover, this analysis was able to confirm the presence of 274 “hypothetical” proteins inferred from gene prediction algorithms applied to the *B. malayi* (Bm) genome. Not surprisingly, the majority (160/274) of these “hypothetical” proteins were predicted to be secreted by Signal IP and/or SecretomeP 2.0 analysis. Of major interest is the abundance of previously characterized immunomodulatory proteins such as ES-62 (leucyl aminopeptidase), MIF-1, SERPIN, glutathione peroxidase, and galectin in the ES of microfilariae (and Mf-containing adult females) compared to the adult males. In addition, searching the ES protein spectra against the Wolbachia database resulted in the identification of 90 Wolbachia-specific proteins, most of which were metabolic enzymes that have not been shown to be immunogenic. This proteomic analysis extends our knowledge of the ES and provides insight into the host–parasite interaction.

## Author Summary

Human lymphatic filariasis caused by the nematode parasites *Brugia malayi* and *Wuchereria bancrofti* are a major cause of concern in tropical countries. Studies over several decades have identified various proteins of these parasites that have highlighted their role in host–parasite interactions and possible chemotherapeutic and prophylactic interventions. The availability of the parasite genome facilitates the identification of all of the proteins of the parasite that could interact with the host. In this study, we have attempted to identify the excretory-secretory proteins of the various stages of the parasite that could be maintained *in vitro* for a limited period utilizing a high-throughput proteomics approach. We observe and report that the parasites expend resources to secrete out various molecules that they utilize to evade the host immune system and modulate its responses. Further, this study also provides information on the predicted hypothetical proteins to be bonafide proteins and thus a catalogue of the excretory-secretory proteins towards a better understanding of the host–parasite interactions.

## Introduction

The parasitic nematodes, *Brugia malayi* and *Wuchereria bancrofti* are lymphatic dwelling filariae that induce a spectrum of clinical manifestations (ranging from clinically asymptomatic [or subclinical] microfilaremia to the deforming elephantiasis) felt to reflect the nature of the filarial specific immune response. The filarial parasite differentiates into a series of morphologically distinct forms in the vertebrate and mosquito hosts during its life cycle, and is comprised of five major stages, separated by 4 molts. The microfilariae undergo two molts in the mosquito before they develop into the infective L3 stage. Once the L3's gain access to the human host they develop over months into adults after 2 molts. After mating, the females release large numbers of microfilariae that move into the blood stream from which they are taken up by the mosquito to complete the life cycle. Each of the filarial life cycle stages has both unique and common biologic characteristics. Because each stage of parasite development may be antigenically distinct, filarial infections are often characterized by a series of discrete immune responses that evolve at different times during the course of infection. Moreover, each stage of parasite development may also entail a change in tissue tropism introducing a compartmental feature to the responses induced by the filarial parasite.

Although the composition of the excretory-secretory (ES) products of the filarial parasites is limited and largely uncharacterized, they have been used as a source in the identification of potential diagnostics [1]–[3] and/or vaccine candidates [4],[5]. Given the potential role of the ES in regulating the host immune system and subsequent pathology associated with the immune response, identifying the individual components of the ES has been of considerable interest. Early studies on the characterization of the ES were limited by both technical/practical constraints along with the low abundance of these proteins often precluding molecular identification [6],[7].

The availability of high throughput and sensitive techniques along with genomic data has facilitated the identification of these proteins. A recent study using comparative analysis of the soluble extracts of mixed adult *Brugia malayi* (BmA) antigen and the excretory-secretory (BES) products of these mixed adults has helped to identify a portion of the secretome contributed by the two adult stages of the parasites [8]. Another study focusing on the stage-specific identification of the ES was reported while this manuscript was under review [9]. The ES data available from these recent studies [8],[9] were based on separation of the ES by gel-electrophoresis techniques followed by LC-MS/MS. The present study utilized a high-throughput, shot-gun proteomic approach to identify comprehensively the secretome of multiple stages of *Brugia malayi* starting with the infective L3 larvae, and moving to those molting to L4, through the adult parasite stages (male and female individually) and to the microfilariae (L1).

## Materials and Methods

### Parasites and *in-vitro* culture

Adult *Brugia malayi* male, female parasites, microfilariae and the L3 larvae were obtained from the Filariasis Research Reagent Repository Center (Athens, Georgia, USA) The adult parasites were washed three times in RPMI media supplemented with antibiotics (100 U/ml penicillin, 100 µg/ml streptomycin, and 0.25 µg/ml of Amphotericin B). The microfilariae were centrifuged at 800×g for 10 mins. The pellet was re-suspended in 10 ml of fresh RPMI-1640 and layered on Ficoll-Hypaque and centrifuged at 1500 rpm for 30 mins. The supernatant was discarded and the pellet containing microfilariae was washed with RPMI-1640. Contaminating RBC were lysed by treating with ACLK solution and washed again. The animal procedures were conducted in accordance with the ACUC guidelines at the National Institutes of Health and at the University of Georgia.

The adult male (1–2 worms/ml), adult female worms (1–2 worms/ml) and microfilariae (0.25×10^6^ mf/ml) were cultured separately in serum-free RPMI 1640 (GIBCO) supplemented with 5 g/L glucose and antibiotic-antimycotic (Invitrogen, 100 U/ml penicillin, 100 µg/ml streptomycin, and 0.25 µg/ml of Amphotericin B). The spent media was collected and replaced with fresh media every 24 to 48 hours to a maximum time of 7 days. The medium collected was filtered through 0.2 µM filters (Millipore) and stored, pooled and concentrated using Amicon Ultrafilters with 3 kDa cut-off membranes. The ES was stored at −80°C until use. Protein concentrations were estimated based on OD_280_ using a Nanodrop ND-1000 Spectrophotometer (Thermo Fisher Scientific, San Jose, CA).

The L3 larvae were cultured as described previously [10] with slight modification. Briefly, 5 to 10 larvae were cultured in 96-well cluster-well plates in 200 µl of serum-free α-MEM supplemented with (ribonucleosides and deoxyribonucleosides), at 37°C with 5% carbon dioxide. The medium was supplemented with penicillin (100 U/ml), streptomycin (100 µg/ml), 0.25 µg/ml amphotericin, 2 µg/ml of ceftazidime and 2 µg/ml of ciprofloxacin. Ascorbic acid (Sigma, St. Louis) was added to a final concentration of 75 µM on day 5 of culture. The media prior to the addition of ascorbic acid was collected every 24–48 hours for analysis of pre-molting ES (L3-ES) and the spent media during and after molting collected as “molting ES (L3-MES)”. The spent media were pooled from various batches, concentrated and subjected to microcapillary reversed-phase liquid chromatography-tandem mass spectrometry (LC-MS/MS).

### Microcapillary reversed-phase liquid chromatography-tandem mass spectrometry (LC-MS/MS)

The ES samples (a minimum of 50 µg) were digested with trypsin at 37°C overnight. The digests were resolved by strong cation exchange LC and analyzed using LC-MS/MS. Microcapillary RPLC was performed using an Agilent 1100 nanoflow LC system coupled online with a linear ion trap-Fourier Transform (LIT-FT) mass spectrometer. Reversed-phase columns were slurry-packed in-house with 5 µm, 300 Å pore size C-18 stationary phase in 75 µm i.d.×10 cm fused silica capillaries with a flame pulled tip. After sample injection, the column was washed for 30 min with 98% mobile phase A (0.1% formic acid in water) at 0.5 µl/min and peptides were eluted using a linear gradient of 2% mobile phase B (0.1% formic acid in acetonitrile) to 40% solvent B in 110 minutes at 0.25 µl/min, then to 98% B for an additional 30 minutes. The LIT-FT mass spectrometer was operated in a data-dependent mode in which each full MS scan was followed by seven MS/MS scans wherein the seven most abundant molecular ions were dynamically selected for collision-induced dissociation (CID) using a normalized collision energy of 35%.

### LC-MS/MS data analysis

Proteins were identified by searching the LC-MS/MS data using SEQUEST against the *Brugia malayi* database downloaded from The Institute for Genomic Research (TIGR) and the *Wolbachia* database from New England Biolabs. Methionine oxidation and phosphorylations on serine, threonine and tyrosine were included as dynamic modifications in the database search. Only tryptic peptides with up to two missed cleavage sites that met the criteria [delta correlation (ΔC_n_)> = 0.08 and charge state dependent cross correlation scores (X_corr_> = 1.9 for [M+H]^1+^, > = 2.2 for [M+2H]^2+^ and > = 3.1 for [M+3H]^3+^] were considered legitimately identified.

### Bioinformatic analysis

For functional annotation of the transcripts, the program Blast× [11] was used to compare nucleotide sequences to the non-redundant (NR) protein database of the National Center for Biotechnology Information (NCBI) and to the gene ontology database [12]. The tool rpsBlast [13] was used to search for conserved protein domains in the Pfam [14], SMART [15], KOG [16] and conserved domain databases [17]. We also compared the transcripts with other nucleotide sequences of *C.elegans*, and Wolbachia. All BLAST comparisons were done with the complexity filter off, but segments of the same nucleotide of 20 bases or larger were masked. To identify possible transcripts coding for secreted proteins, the BMA-pep sequences (obtained from TIGR *Brugia malayi* database), were submitted to the SignalP server [18] to identify translation products that could be secreted. In addition, non-classical secretion analysis was carried out using the SecretomeP2.0 [19]. Transmembrane helices were predicted using the TMHMM program [20]. Functional annotation of the transcripts was based on all the comparisons above and manually curated. Following inspection of all these results, transcripts were classified into various functional classes, with further subdivisions based on function and/or protein families. The entire annotated database (with embedded links) is accessible at http://exon.niaid.nih.gov/transcriptome/Bm-secretome/Brugia-Secretome-Web.zip. A standalone version can also be accessed and downloaded at http://exon.niaid.nih.gov/transcriptome/Bm-secretome/Brugia-Secretome-SA.tar.gz.

### PCR

DNA was isolated from the culture media of parasites and used to amplify with primers (wsp forward primer: GATGAGGAAACTAGTTACTA; wsp reverse primer: CCAAATAGCGAGCTCCAGC) targeting the Wolbachia wsp gene. As positive controls, genomic DNA isolated from *Brugia malayi* and *Wuchereria bancrofti* were used.

## Results

### *Brugia* secretome overview

**Figure 1:**
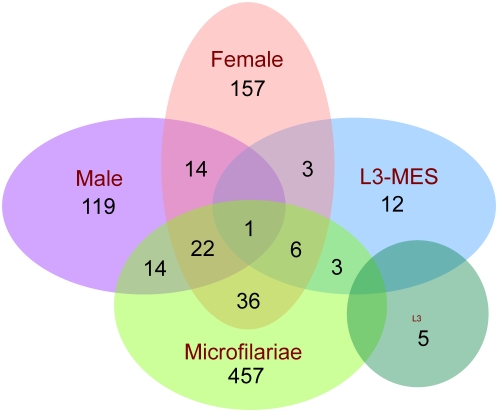
Stage specific distribution of ES in the adults, microfilariae, L3 larvae and L3 larvae molting to L4 of *Brugia malayi*. Proteins identified are represented as a Venn diagram, illustrating the overlap in proteins detected in the adult male, adult female, microfilarial and larval stages of *B.malayi*. The numbers indicate the total number of proteins detected in each of the stages. The complete listing of each of these proteins along with the peptides identified are given in Table S2, Table S3, Table S4, Table S5, and Table S6.

Analysis of data from the filarial genomic project indicated that 1,022,933 peptides could theoretically be generated from tryptic digestion of the 11,610 predicted proteins of *Brugia malayi* [21]. Our proteomic analysis revealed the *Brugia malayi* secretome to be surprisingly complex. The identified ES proteins can be found in concise form in Table S1 and in a complete annotated table with embedded links at Final Secretome-Web (http://exon.niaid.nih.gov/transcriptome/Bm-secretome/Brugia-Secretome-Web.zip). From a total of 1040 unique and 65 non-unique (defined as peptides that matched 100% with more than one filarial protein) peptides identified from the adult male, adult female and microfilarial stages of the parasite, 170, 239 and 540 proteins, respectively were identified (Figure 1). Interestingly, the ES products of the L3 larvae was limited to the identification of unique peptides that matched only 5 proteins with another 25 proteins being identified in the L3/L4 molting stage (L3-MES). In addition, non-unique peptides (to which a definitive protein match could not be made) identifying 9 proteins from adult males and 12 from the adult females, 36 proteins from microfilariae and 3 proteins each from the L3-ES and L3/L4 ES (L3-MES) were detected (Table S2, Table S3, Table S4, Table S5, and Table S6, highlighted in yellow). Of the 852 distinct (or different) proteins identified in the secretome, 215 (25.2%) would have been predicted based on clustered expressed sequence tagged (EST) sequences. Among the 852 proteins identified in the ES products, 274 are annotated as ‘hypothetical’. The identification of these “predicted proteins” as clearly being measurable proteins provides validation that these are *bona fide* gene products. Moreover, each of these 274 proteins have orthologues in *C.elegans* (Final Secretome-Web) (http://exon.niaid.nih.gov/transcriptome/Bm-secretome/Brugia-Secretome-Web.zip).

Comparative analysis of these 852 proteins between the secretome and the somatic proteome (unpublished) of *Brugia malayi* suggest that 118 were exclusive to the ES products, of which 89 (72%) were hypothetical proteins or uncharacterized proteins with no known function.

On a weight per volume basis, male adult parasites (about 20% of the female body weight) produced 8–9% of the ES produced by an adult female. The female parasites were found to excrete/secrete not only more protein per worm but also a greater number of proteins, many of which have previously been shown to be gender specific [7].

### Stage specificity in the proteome

The overwhelming majority of secreted molecules were stage-specific (Figure 1). As can be seen, 119/170 (70.0%) of the ES products of the adult males, 157/239 (65.6%) of the ES products of the adult females, 457/540 (84.6%) of the ES products of the microfilariae, and 12/27 (44%) and 5/8(62.5%) molting L3-ES and L3-ES respectively, were not shared between or among the stages studied.

### Secretory analysis

Fifteen percent of the 852 proteins identified within the ES were predicted to contain either 5′ signal peptide sequences or signal anchors. However, ∼50% of the total number of ES proteins of *Brugia malayi* predicted to be non-secretory by SignalP, would be predicted to be secretory by Secretome P. (Final Secretome-Web) (http://exon.niaid.nih.gov/transcriptome/Bm-secretome/Brugia-Secretome-Web.zip). Combining the results obtained using both methods, results in 54.5% (437/802) of the proteins being predicted as having the necessary motifs to be secreted.

### Comparison to previous analyses of ES products

Using conventional biochemically-based methods approximately 20 secreted or excreted proteins of *Brugia malayi* and other related parasitic nematodes have been identified [22]–[35]. Our analyses were able to corroborate the overwhelming majority of these except Bm20, BmTGH-1, GST, cathepsin, and SXP-1. More recently, higher throughput methods have been applied to characterize the proteomes of mixed adult males and females [8] or of adults and microfilariae [9], Our study identified 40 of the 80 proteins identified in Hewitson et. al. [8] and 90 of the 228 identified by Moreno et.al [9], but failed to detect the remaining proteins that include protein disulphide isomerase (PDI), BmR1, and tropomyosin. In addition we were able to identify some of the proteins such as phosphatidylethanolamine binding proteins, phosphofructokinase and cyclophilin-5 that were detected by Hewitson et.al., [8] and could not be found in the study by Moreno and Geary (9). Moreover, in these two stages (adult females and males cultured separately), we were able to identify an additional 320 proteins, listed in Table S1 and in Final Secretome-Web (http://exon.niaid.nih.gov/transcriptome/Bm-secretome/Brugia-Secretome-Web.zip).

### Functional annotation

**Figure 2:**
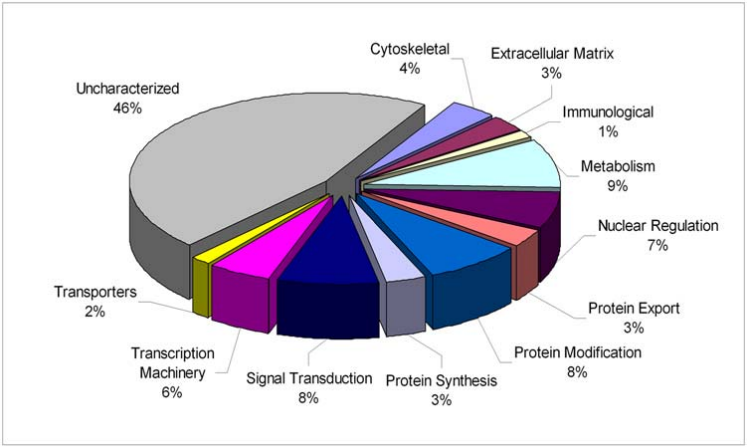
Functional profiles of *Brugia malayi* ES proteins. Pie-chart representing the percentage of proteins identified from each of the stages, plotted based on their functional classification. Only a single annotation was assigned to a given protein. All unknown and hypothetical proteins have been classified as uncharacterized. Note: Metabolism includes amino acid, carbohydrate, nuclear and energy metabolism. A complete list is given with additional annotation and embedded links in the Final Secretome-Web (http://exon.niaid.nih.gov/transcriptome/Bm-secretome/Brugia-Secretome-Web.zip) version. *AMES*: Adult male ES; *AFES*: Adult female ES; *MFES*: Microfilariae ES; L3-ES: L3 larval ES; L3-MES:L3 larval molting ES.

**Figure 3:**
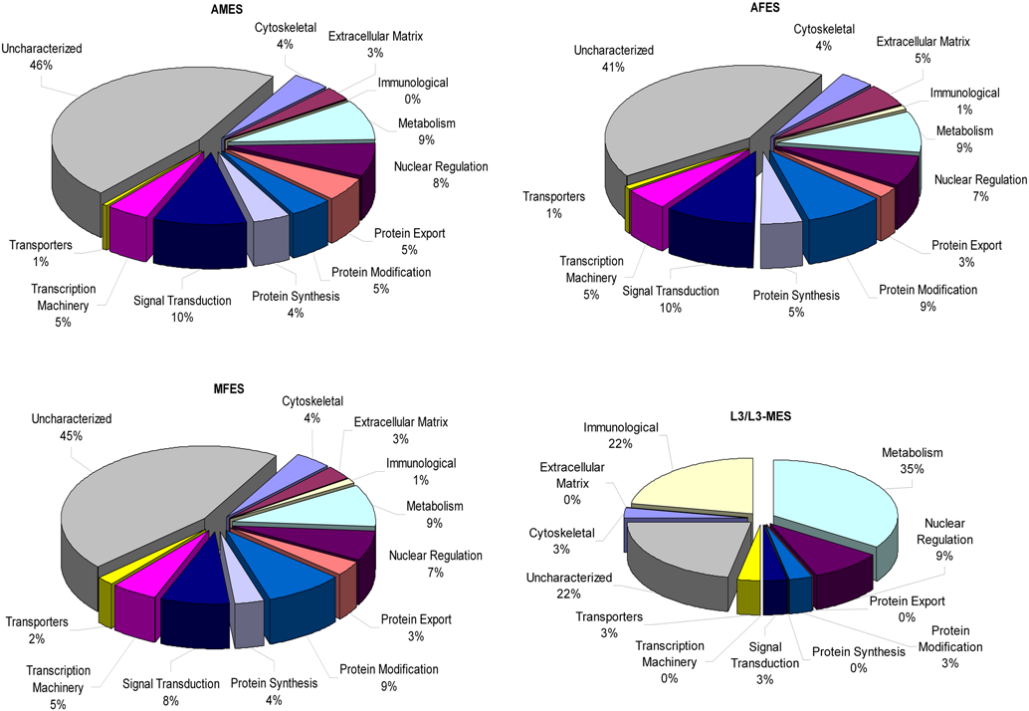
Stage specific functional profiles of *Brugia malayi* ES proteins. Pie-charts representing percentage of proteins identified from each of the stages, plotted based on their functional classification. Only a single annotation was assigned to a given protein. All unknown and hypothetical proteins have been classified as uncharacterized. Note: Metabolism includes amino acid, carbohydrates, nuclear and energy metabolism). The complete list is given with additional annotation and embedded links in the Final Secretome-Web (http://exon.niaid.nih.gov/transcriptome/Bm-secretome/Brugia-Secretome-Web.zip) version. *AMES*: Adult male ES; *AFES*: Adult female ES; *MFES*: Microfilariae ES; L3-ES: L3 larval ES; L3-MES:L3 larval molting ES.

Proteins were classified into main functional classes based widely on the KOG classification of the *C.elegans* orthologues from Wormbase release WS184, with some adaptations for uncharacterized protein group comprising of hypothetical or uncharacterized conserved or unknown proteins. Metabolic processes involved in carbohydrates, amino acids, nuclear and energy were collectively classified into the category of metabolism. From the entire annotated secretome (Figure 2), 46% of the proteins do not have any known function and/or are predicted hypothetical genes. As mentioned above, these data emphasize that not only are the predicted hypothetical proteins actually being synthesized, but they might also play an important role at the host parasite interface. When examined by parasite stage, it is clear that none of the stages appeared to be biased toward a particular set of functional processes (Figure 3). A complete list of the functionally classified proteins is given in Final Secretome-Web (http://exon.niaid.nih.gov/transcriptome/Bm-secretome/Brugia-Secretome-Web.zip).

### Abundance of ES proteins

Spectral counts have been adopted to reflect the relative abundance of individual proteins [36]. The number of times a particular peptide is seen in the spectra (Spectral Counts) can be correlated with abundance of that particular protein. The abundance does not represent a true molar concentration of the proteins in the ES, but rather serves to differentiate between highly abundant proteins from those of lesser abundance. The number of peptides and the peptide(s) identified for each protein in the various stages are listed (Table S1, Table S2, Table S3, Table S4, Table S5, and Table S6).

### Abundance—L3 to L4 infectome

The transition of L3s to the L4 stage reveals the identification of several immunologically important parasite proteins. Analysis of the proteins secreted by the L3 larvae indicates an abundance of the abundant larval transcripts (ALT-1 and ALT-2; Bm1_26880, Bm1_28735) that are not detected after the L3's molt to L4s. Although cysteine proteases (i.e., cathepsins) have been implicated in molting of the L3 to L4 stage [37],[38], we were not able to identify these proteins in the secretome irrespective of the stage examined.

The *Brugia malayi* homologue of LL20 (Bm1_50995), which has been identified as a nematode polyprotein allergen (NPA), is an immunologically important antigen that is comprised of subunits that form a ladder 15 kDa increments on SDS-PAGE gels. Peptides matching to LL20 were found to be abundantly secreted not only by the molting L3s but also by the adult females and the microfilariae (Table S1, Table S3, and Table S6). Another molecule that was found to be abundantly secreted only by the molting L3s is the DJ-1 family protein (Bm1_07685).

Among several of the parasite proteins identified in the ES that were “non-unique” was the larval allergen (Bm1_17875) known to be among the most abundantly released by the L3 larvae and, by sequence analysis, is related to the ALT family of proteins. The larval allergen (see Table S5) forms the majority of the L3-ES.

**Figure 4:**
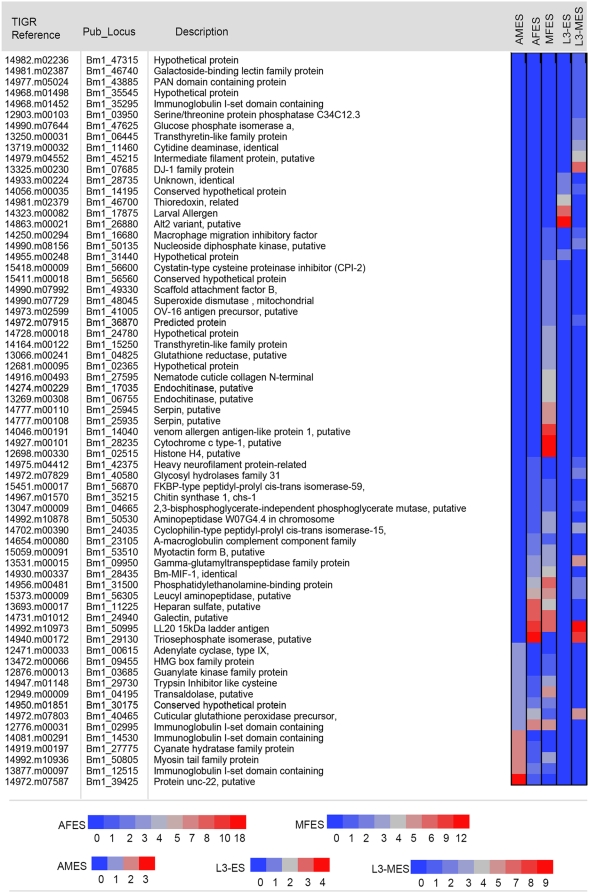
Relative abundance of ES proteins of *Brugia malayi*. The most abundant proteins identified (relative abundance of peptides) of the *Brugia malayi* ES of adult females, adult males microfilariae, and L3 larvae (pre- and during molting) are illustrated as a heat map. Relative abundance scale are coded (spectral values) based on number of peptides identified per protein with separate scales for each stage on the bottom of the figure. *AMES*: Adult male ES; *AFES*: Adult female ES; *MFES*: Microfilariae ES; L3-ES: L3 larval ES; L3-MES:L3 larval molting ES.

Parasite encoded stress-inducible secretory proteins such as thioredoxin peroxidases, superoxide dismutases, glutathione peroxidases have the capacity to neutralize the reactive oxygen species and form a potential immune evasive strategy for parasite survival. During the molting stage of the L3 larvae, thioredoxins (TRX; Bm1_46700, Bm1_46705) and glutathione peroxidases (GPX; Bm1_40465) were found to be secreted (Figure 4 and Table S6). Analysis of the ESTs for GPX shows that the L4 stage showed comparatively higher gene expression than in L3 (L3 = 0; L4 = 7).

Another protein found to be abundant in the ES at the time of the L3/L4 transition and not in any of the other stages examined is the intermediate filament protein (Bm1_45215), that has orthologues in *O. volvulus* (Ov1CF or OV-IF), *A. lumbricoides* IF-A and *C .elegans* (IFA-1). IFA-1 is required for survival past the L1 stage and is predicted to function as a structural component of the cytoskeleton. In addition, several proteins known to modulate the immune system of the host, leucyl aminopeptidase (ES-62) [39], gamma-glutamyl transpeptidase (γ-GT) [40], macrophage migration inhibitory factors (Bm-MIF) [29],[41], and the galectins [42], were detected in the molting ES during the L3/L4 molt.

### Abundance—Adults and microfilariae

Compared to the L3 and L4 larvae that interface with the host over a relatively short period, the adult and microfilarial stages provide at both a quantitative and temporal level the overwhelmingly abundant levels of ES proteins. Several extracellular proteins including collagens, ankyrins, cuticulins and other associated proteins; and cytoskeletal proteins such as myosin and kinesin-like molecules could be detected. Galectins (Bm1_46740, Bm1_24940) or galactoside binding lectins, have been found to be abundant in the ES (Figure 4), and by inference could influence the host immune response and modulate (or mediate) pathologic responses.

A number of enzymes involved in the general metabolism of parasites have been proved to be immunologically important. Gamma glutamyl transpeptidase (γ-GT – Bm1_09950) is a multifunctional enzyme involved in the metabolism of glutathione and has been found to be released by the adult females and microfilariae but not adult males. γ-GT specific IgE antibodies appear to be associated not only with tropical pulmonary eosinophilia but also possible resistance to filarial infections [40]. This enzyme could also be involved in the catabolism of immunomodulatory and pro-inflammatory cysteinyl leukotrienes [43]. Triose-phosphate isomerase (TPI – Bm1_29130) an enzyme involved in the conversion of dihydroxy-acetone to glyceraldehyde-3-phosphate was specifically identified abundantly in the ES of the adult female, and not in the adult male or microfilariae. TPI has been previously reported in the secretions of *Schistosomes* and *Haemonchus contortus*, suggesting it has an important, but yet unidentified role, in host-parasite interactions. Previous studies on the Brugia secreted products also found TPI to be the most abundantly expressed protein by the adults and microfilariae [8],[9]. Incidentally, TPI is the most abundant protein in the ES based on relative abundance of unique peptides identified not only in the adult females but also in the molting L3 larvae (Figure 4). The other glycolytic enzymes detected at lower abundance levels are enolase (Bm1_24115) and transaldolase (Bm1_04195). While transaldolase was maximally observed in the secretions of microfilariae and sparingly in adults, enolase was detectable only in the secretions of the adult male parasites.

### Immune modulation

Previously identified immunomodulatory proteins such as BmMIF-1 (Bm1_28435) and ES-62 (Leucyl aminopeptidase, Bm1_56305) were found to be abundant in the ES of adult females and microfilariae. Among the cytokine homologues of the parasite (macrophage migration inhibitory factor (MIF) and TGF-β), only Bm-MIF was identified in high abundance in the microfilariae and the adult females. Based on the abundance levels, it is not clear whether these proteins are the secretions of the uterine microfilariae or the adult female *per se*. At the transcriptional level, the females have a 2.4 fold increased expression of BmMIF -1 than in the adult males (data not shown). In addition, MIF–2 (Bm1_16680) was detected in the ES of the molting L3 (L3-MES). ES-62 or leucyl aminopeptidase is a phosphorylcholine containing glycoprotein that is secreted only by the post infective larvae in *A. viteae*. Similar to *A. viteae*, the ES-62 homologue from *Brugia malayi* was found to be secreted by the L3s molting to L4s (Table S1 and Table S6).

Of the surface associated proteins, the anti-oxidant products glutathione peroxidase (GPX, Bm1_40465)) and superoxide dismutase (SOD, Bm1_48045) are important proteins suggested to be involved in immune evasion that have been detected in the microfilariae and the adults parasites. Abundance analysis indicates that SOD is more specific to the ES of microfilariae compared to adults, while GPX could be detected in each of the parasite stages with molting L3/L4 larvae and microfilariae containing greater amounts than the adults (Figure 4).

In a similar fashion, the cystatins (CPI-2, Bm1_56600) and serpins (Bm1_25935, Bm1_25945, and Bm1_28525) have been found to be released in greater amounts by microfilariae compared to adults. The most abundant microfilarial transcript from *Brugia malayi* encodes serine protease inhibitors (serpins) and was found only in the microfilarial stage. Another protease inhibitor-like molecule that was prominently identified is the phosphatidyl-ethanolamine binding protein (Bm1_31500, Bm1_41005). It is not yet clear if the parasite encoded or phosphatidyl-ethanolamine binding proteins or the orthologues from other helminths (Ov-16) have protease-inhibitory activity.

One of the proteins of interest that was identified was the vespid venom allergen homologue-like (VAH; Bm1_14040) protein that was detected in the ES of microfilariae. Peptides matching the microfilarial sheath protein (Bm-SHP3; Bm1_50600, Bm1_50585) were also found to be secreted by the microfilariae alone and not by the adult parasites.

Endochitinase (Bm1_06755, Bm1_17035) and nematode cuticle collagens (Bm1_27595) were found to be released in greater amounts by the microfilariae compared to the adults. Results from the secretome analysis indicate the presence of chitinases (CHI-1 and CHI-2) only in the microfilarial stages including the uterine/new born microfilariae (unpublished). Nematode cuticle collagens and its associated enzymes that are involved in cuticle synthesis (peptidyl-prolyl cis-trans isomerase (PPI, cyclophilins (Bm1_24035) / FKBP (Bm1_35815, Bm1_56870)), prolyl-4-hydroxylase (PHY), cyclophilins and FKBPs) were easily detected in the secretome.

### Wolbachia proteins

**Figure 5:**
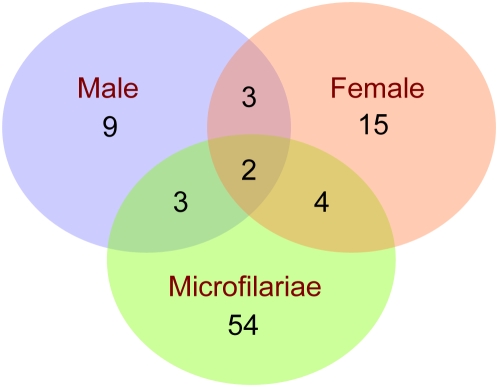
Distribution and functional classification of *Wolbachia* ES proteins. Proteins identified in the ES were searched against the Wolbachia database and a total of 91 were identified in the ES of *Brugia malayi*. The pie-chart represents the percentage of Wolbachia proteins identified from all of the stages, plotted on as a function of their classification. Only a single annotation was assigned to a given protein. All unknown and hypothetical proteins have been classified as uncharacterized. (Note: Metabolism includes amino acid, carbohydrates, nuclear and energy metabolism.)

In the present study 90 proteins (listed in Table S7) of Wolbachia origin were found in the ES of the adults and the microfilariae, with the microfilariae contributing maximally. Single peptide hits matching Wolbachia proteins formed a majority (68 proteins) of the proteins identified in the adult ES, while the Wolbachia proteins represented by more than one peptide seemed to be involved in nuclear metabolism (based on functional annotation). In contrast, the Wolbachia proteins were not detectable in the infective L3 larvae or the molting larvae. Classification of the Wolbachia proteins did not result in the identification of any particular class of proteins that could be mapped to specific known biochemical pathways. Functional annotation of the Wolbachia proteins in the ES suggests a wide range from uncharacterized proteins, as well as those involved in nuclear regulation, metabolic enzymes, protein synthesis and modification (Figure 5).

Further, to evaluate if Wolbachia themselves were released into the culture media [these intracellular bacteria being a surrogate for potential death of the *in vitro* cultured parasites], DNA isolated from the ES of microfilariae, adult males and females was assayed for Wolbachia surface protein (wsp) gene by PCR. First, there was only a very minimal amount of DNA present in the culture media based on 260/280 nm ODs; secondly what little DNA there was did not contain Wolbachia (Figure S1).

## Discussion

Parasitism, when successful, requires genetic adaptations to permit survival within a potentially hostile host. While the adult parasites of *Brugia malayi* reside in the lymphatics, microfilariae circulate in the blood, where they are bathed in immune and other effector molecules. As such, the parasites must secrete biologically active mediators that modify or customize their niche within the host to survive. Such secretions have been the focus of previous biochemical and immunological analyses [44],[45]. Data from expressed sequence tag (EST) analysis identified filarial proteins mimicking those of cytokines, chemokines and other immune effector/modulatory molecules of humans [24],[29]. These proteins have been predicted to or shown to promote parasite survival or development, and suggests that the parasites, by secreting or expressing these on surface exposed membranes, have adapted these gene products to promote successful parasitism.

ES proteins contain proteins actively shed from the parasite surface as well as those secreted from the pharyngeal glands, the excretory system, epithelial cells of the intestine (e.g. digestive enzymes) and/or rectal and vaginal cells [46]. Though the requirement of a signal peptide was thought to be crucial for proteins to be secreted, many extracellular proteins can, however, be exported without a classical N-terminal signal peptide [47]–[49]. The conversion of gene products (Bm-SPN2 [50], MIF [29]) in *Brugia malayi* or OvGST [51] in *Onchocerca volvulus* to secretory proteins may be a common adaptive strategy for parasitic organisms.

It can only be speculated that over 46% of the identified secreted proteins not containing predicted secretory signals/features are being excreted by alternate mechanisms (Secretome P2 [19]) and might reflect on the limitation of the algorithms ‘trained’ on non-nematode sequences. The identification of “predicted” or “hypothetical proteins in the proteome analysis validates these as being *bona fide* gene products. These gene products are of interest for their filarial-specific nature and warrant functional characterization of their role in parasite and host-parasite interactions. Many of these proteins could also serve as targets for drugs and prophylactics as suggested previously [52].

There were 118 proteins that were detected only in the ES, but not found to be represented in the somatic proteome (unpublished). It is not yet clear how or why these proteins were found only in the ES though it is possible that these proteins are present in relatively low amounts in somatic protein preparations but are enriched in the ES products. Similar results have been found when the ES proteins were compared to the somatic extracts of *Brugia malayi* [8]. In that same study, about 80 proteins were identified in the secretome of mixed adults by LC-MS/MS as ES in nature, with an additional 63 proteins in the soluble somatic extracts of *Brugia malayi* (BmA) [8]. By utilizing a more sensitive (non gel-electrophoresis) approach we were able to identify a number of proteins of low abundance that were not detectable in the previous studies [8],[9]. Indeed, many of the proteins identified in the present study were also identified in the other two studies [8],[9]. Our data, extends the analyses to the L3 and molting L3/L4s in addition to the adults [8],[9] and the microfilariae [9]. It is possible that the ES analysis among the various stages might vary (study to study) based on the exact experimental conditions, batch to batch processing and as a consequence of immunologic (or other factors) that occur *in vivo.*

### Stage specificity

Since the filarial parasites develop through multiple stages, how the different stages orchestrate the production of ES proteins becomes important for understanding successful parasite development and survival. Interestingly our data suggest that the filarial parasites utilize many secretory proteins in an as yet unknown but stage specific manner.

The identification of ALT proteins only in the infective L3 larval stage corroborates previous EST analyses [53]. Furthermore, the importance of ALT proteins in invasion [54],[55] suggests a primary parasitic adaptation by secreting molecules that help in the establishment of the infection.

An interesting and intriguing aspect of the present study is the observation that the microfilariae appear to secrete/excrete more (at a quantitative level and weight/volume basis) proteins than do the adult stages. Some of these proteins identified in this stage, such as the serpins, had previously been shown to be microfilarial-specific [56]. Interestingly, microarray analysis indicated a 4-fold upregulation of the serpins in the adult females (containing uterine microfilariae) compared to the adult males (data not shown). Among the protease inhibitors, CPI-2 is constitutively expressed transcriptionally by all stages of the parasite life cycle [27],[57],[58], though, in the present study, the CPI-2 protein was found to be secreted only by the microfilariae. Interestingly, expression profiles of CPI-2 [57] have shown CPI-2 mRNA expression to be higher in L1 (microfilaria) and L3s compared to the adults stages [27],[58]. Like the serpins, endochitinases are microfilarial specific proteins that are absent in the adult worms, with synthesis starting in newborn microfilariae 2 days after birth [59]. The possible functional role played by these chitinases have been reviewed previously [60] but appear to be restricted to the microfilarial stage.

### Molting

Recent advances in the understanding of the molting process bring into light the roles for several peptidases and specifically the metallo-peptidases that may be potential candidates for chemotherapeutic interventions [61]–[63]. In this study, apart from leucyl aminopeptidase (ES-62) we were unable to identify other aminopeptidases in the molting L3 ES. Though cathepsins have been implicated in the molting process, they were not detected in the ES of molting L3 larvae. Transcriptional analysis of the molting process, currently underway, should address this more conclusively.

### Cuticles

Although each stage of the parasite has a cuticle, it is apparent that the microfilariae expend a large amount of energy in the maintenance and generation of their cuticular components (e.g. peptidyl-prolyl cis-trans isomerase (PPI, Cyclophilins (Bm1_24035) / FKBP (Bm1_35815, Bm1_56870)), prolyl-4-hydroxylase (PHY). Cyclophilins (CYPs) and FKBPs are large multi-member families in nematodes [64]. Representative members of the ‘cyp’ genes have been identified in *B.malayi* and other parasitic nematodes [64]–[69] as well. Whether these parasite-derived molecules function as human homologues is unknown.

### Immune evasion and immunomodulation

The success of a parasite to establish infection depends on its abilities to subvert the host immune defenses and survive in the host for a long time without eliciting an inflammatory reaction. In the current study we have identified a number of proteins that have been implicated such as the abundant larval transcripts, thioredoxins, and glutathione peroxidases in evading the host immune responses. Analyses of the anti-free radical defenses of *Brugia malayi* indicate the parasite can secrete GPX, TRX and SOD. Glutathione peroxidases (GPX) are surface associated proteins that protect the parasite by neutralizing the reactive-oxygen intermediate attack, or modifying and removing host immunomodulatory lipids. Thioredoxins (TRXs) are a large family of anti-oxidant proteins produced by a wide range of organisms in defense against toxic hydroxyl radicals that damage proteins, lipids and DNA. Despite the lack of a signal sequences, thioredoxins are secreted by the parasites and were only found in the ES of the molting L3's. Together with GPX, TRX provides the parasite with essential defense against reactive oxygen species. In the current study, superoxide dismutase (SOD) was been found to be present most abundantly in the ES of the microfilariae, and finding that differs to that which has been described previously [70].

Though DJ-1 was found to be secreted by the mixed adult parasites [8] and in the adults and microfilariae [9], our study identified this protein to be abundant in the molting L3 larval secretions. Very little is known about the functional role of DJ-1 except its association with Parkinson's disease [71]. The multitude of functional groups within the DJ1/ThiJ/PfpI family makes it difficult to infer any specific role of the protein in the parasite. Analysis of homologous sequences to DJ-1 suggests that DJ-1 may have a role in thiamine metabolism. However, the association of DJ-1 with chaperones [72],[73] and its KOG classification (KOG2764) suggests a particular role for it in evading the host immune system.

Although the LL20 ladder protein (NPA) was found to be abundantly present in the ES of the molting L3's and the adult female, interest in NPAs is not limited to their abundance but extends to their intrinsic role as potential allergens [74] and their potential role in the sequestration of immunologically important signaling lipids that can, in turn, modulate local host inflammatory reactions [74],[75].

Several known immunomodulatory molecules of filarial parasites were identified in the ES in the present study. Among them were ES-62, serpins, galectins and MIF-1. ES-62 has been shown to modulate key signal transduction pathways associated with immune cell activation and polarization [76]. Leucine aminopeptidases have also been implicated in the metabolism of cysteinyl leukotrienes as has gamma-glutamyl transpeptidase [43]. Given the abundance of ES-62 and gamma glutamyl transpeptidase in the secretome of the parasite their role in modulating lymphatic (and vascular) endothelial cells exposed to these proteins *in situ* is of obvious importance.

Another major component of the *Brugia malayi* secretome were the galectins. Galectins are atypical secreted proteins that occur both extra- and intracellular and have been identified from a number of filarial parasites [77]–[79]. Accumulation of galectins occurs at endothelial interfaces by binding to extracellular proteins and promoting endothelial proliferation [80]. The ability of galectins to bind specifically to IgE [78], to regulate alternative macrophage activation [81], and inhibit lymphocyte trafficking [82] suggests additional potential roles for these proteins.

Another interesting but unexplained feature of the ES products is the identification of cytochrome C (Bm1_28235) and histone-like molecules. These proteins are amongst the most abundant proteins of the *Brugia malayi* secretome (Table S1). Studies on the ES proteins of other parasites [83],[84] have also identified these molecules, though no specific functional implications have been attributed to them. Similar findings of nuclear, cytoskeletal and mitochondrial proteins in the two recently published studies [8],[9] in the ES proteins of the filarial parasites suggest that this is due to the active release of the proteins and not due to chance identification of these proteins contaminated by dying worms.

Interactome analysis of the *B.malayi* ES products, based on the *C.elegans* interactome was unable to demonstrate networks of interacting proteins (data not shown).

### Wolbachia

The Wolbachia endosymbiont is very essential for Brugia worms, as antibiotic treatment that targets the Wolbachia results in the loss of adult worms viability [85]. Molecules of Wolbachia origin released by the parasite has been postulated to influence the inflammatory responses associated with infection [86]. Microfilarial ES contained a majority of the Wolbachia proteins identified. Most of these proteins were present in extremely low amounts in the ES.

It is possible that dying parasites or disintegrating embryo's shed out by the adult worms could release Wolbachia proteins. In our system at least, though there were no dead adult or larval worms in culture, dying microfilariae could have resulted in the identification of some of the low abundant Wolbachia proteins. Wolbachia DNA, however, could not be detected in highly concentrated microfilarial ES (Figure S1) suggesting that the gene products were indeed exported from their intracellular location. It is possible that LC-MS/MS data acquired of spots excised from the 2D-gels or 1D-gels [8],[9] followed by LC-MS/MS was unable to identify these very low abundance proteins.

### Conclusions

This study provides a comprehensive examination of the secretome from most stages of the *Brugia malayi* parasite. The secretome is comprised of a large array of proteins many of which have been shown previously to modulate (either directly or indirectly) the host immune system, potentially promoting their survival. In addition, many “hypothetical proteins” have been detected whose presence provides independent corroboration of the Brugia Genome Project's gene prediction algorithms. The application of high-throughput proteomic techniques in the secretome analyses resulted in the identification of low abundance proteins that might not be detected using gel electrophoresis-based methods. Typical SignalP analyses could not account for the most of the ES products and suggests that new methods for these predictions need to be tailored to helminths. It is expected to have variations in the secretion profiles when the parasites are exposed to the host immune attack. Thus, this *Brugia malayi* secretome, in addition to the previously published secretomes of the filarial parasite *Brugia malayi* [8],[9], should help enable the study of the host-parasite interface in much greater detail than has been possible heretofore.

## Supporting Information

Figure S1 Wolbachia DNA is absent in the parasite cultures. A gel-like image from the Agilent Bioanalyzer 2100 of amplified PCR products of DNA made from culture media from microfilariae, adult males and adult females, using primers to the Wolbachia wsp gene. Lane 1- Negative control, Lane 2 - DNA from MF culture media, Lane 3 - DNA from adult male culture media, Lane 4 - DNA from adult female culture media, Lane 5 - genomic DNA of B.malayi, Lane 6 - genomic DNA from *W. bancrofti*.
(0.75 MB TIF)

Table S1 Annotated secretome. Proteins identified in the ES of *Brugia malayi* are listed as spectral values (abundance / number of peptides identified per protein). The Pub-Locus is a stable gene ID. Reference ID is the TIGR reference gene ID, AMES is the adult male ES, AFES is the adult female ES, MFES is the microfilarial ES, L3-ES is the L3 larval ES, L3-MES is the molting L3 ES. * denotes that the peptides identified are non-unique (highlighted in yellow), but mapped to a protein family e.g. Thioredoxins.; N#Y followed by a number denotes the Y-number of non-unique peptides (highlighted in light green) identified for that particular protein with N-number of unique peptides identified. A fully annotated secretome is given in Final Secretome-Web (http://exon.niaid.nih.gov/transcriptome/Bm-secretome/Brugia-Secretome-Web.zip)
(0.17 MB XLS)

Table S2 AMES Peptide List. The table describes the peptides identified and the corresponding protein in the ES of adult male *Brugia malayi*. NTPS - Number of times peptide seen; NSH - Non-specific hits (Peptides matching with more than one protein; the number denotes number of proteins that the peptide has matched hits by BLASTP). Non-specific peptides are highlighted in yellow.
(0.07 MB XLS)

Table S3 AFES Peptide List The table describes the peptides identified and the corresponding protein in the ES of adult female *Brugia malayi*. NTPS - Number of times peptide seen; NSH - Non-specific hits (Peptides matching with more than one protein; the number denotes number of proteins that the peptide has matched hits by BLASTP). Non-specific peptides are highlighted in yellow.
(0.10 MB XLS)

Table S4 MFES Peptide List. The table describes the peptides identified and the corresponding protein in the ES of microfilariae of *Brugia malayi*. NTPS - Number of times peptide seen; NSH - Non-specific hits (Peptides matching with more than one protein; the number denotes number of proteins that the peptide has matched hits by BLASTP). Non-unique peptides are highlighted in yellow.
(0.19 MB XLS)

Table S5 L3-ES Peptide List. The table describes the peptides identified and the corresponding protein in the ES of *Brugia malayi* L3 larvae. NTPS - Number of times peptide seen; NSH - Non-specific hits (Peptides matching with more than one protein; the number denotes number of proteins that the peptide has matched hits by BLASTP). Non-unique peptides are highlighted in yellow.
(0.05 MB XLS)

Table S6 L3-MES Peptide List. The table describes the peptides identified and the corresponding protein in the ES of *Brugia malayi* L3 larvae molting into L4 stage. NTPS - Number of times peptide seen; NSH - Non-specific hits (Peptides matching with more than one protein; the number denotes number of proteins that the peptide has matched hits by BLASTP). Non-unique peptides are highlighted in yellow.
(0.06 MB XLS)

Table S7 Wolbachia ES. List of proteins in the secretome being identified as Wolbachia in origin.
(0.05 MB XLS)
