# Supplementary figures and images for: Brugia malayi Excreted/Secreted Proteins at the Host/Parasite Interface: Stage- and Gender-Specific Proteomic Profiling
Source: PLoS Negl Trop Dis. 2009 Apr 7;3(4):e410. doi: 10.1371/journal.pntd.0000410 (PMC2659452; doi:10.1371/journal.pntd.0000410)

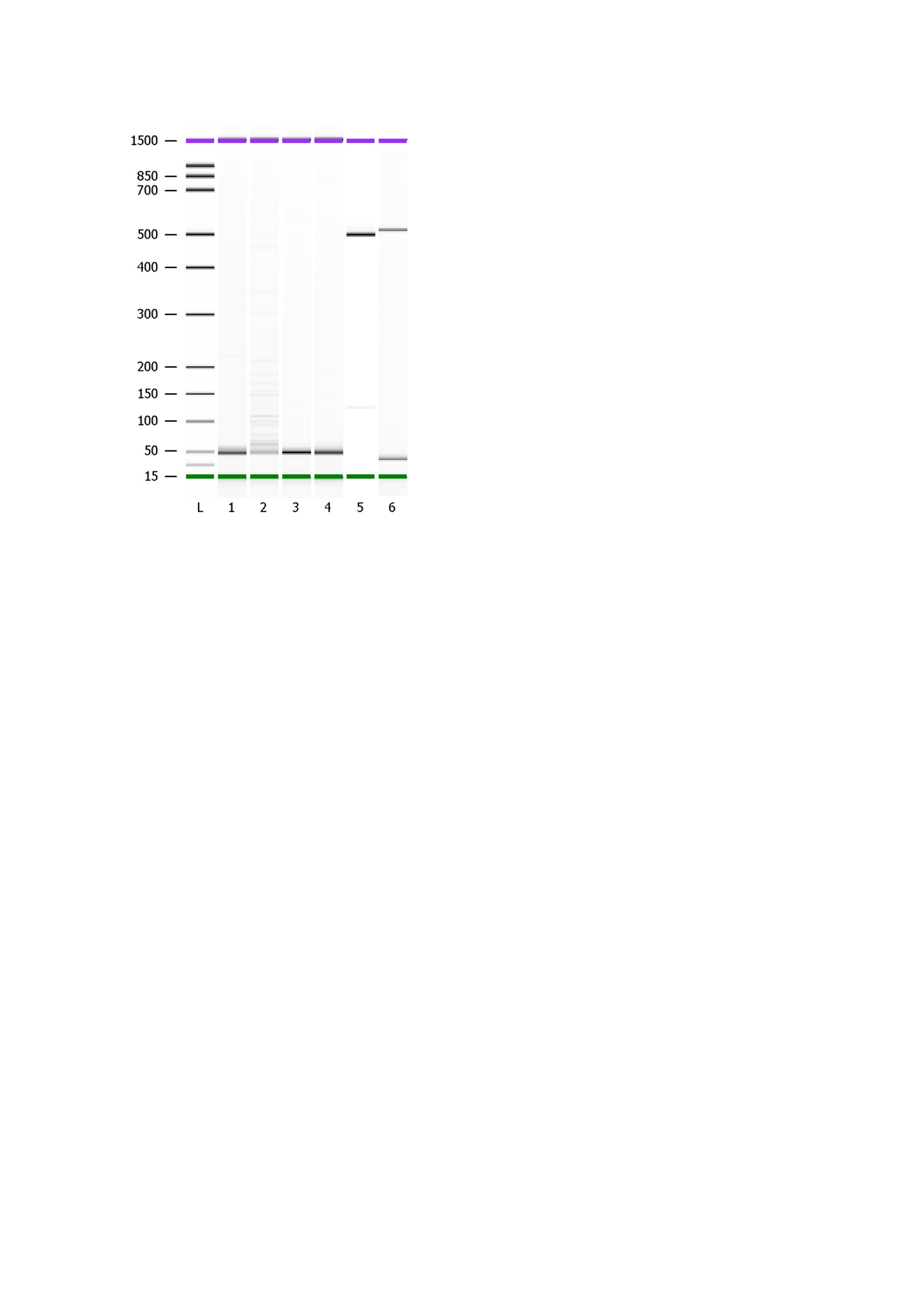

Supplement: Figure S1 — Wolbachia DNA is absent in the parasite cultures. A gel-like image from the Agilent Bioanalyzer 2100 of amplified PCR products of DNA made from culture media from microfilariae, adult males and adult females, using primers to the Wolbachia wsp gene. Lane 1- Negative control, Lane 2 - DNA from MF culture media, Lane 3 - DNA from adult male culture media, Lane 4 - DNA from adult female culture media, Lane 5 - genomic DNA of B.malayi, Lane 6 - genomic DNA from W. bancrofti. (0.75 MB TIF) [file pntd.0000410.s001.tif]
